# Prognostic Value of Non-Contrast CT Markers and Spot Sign for Outcome Prediction in Patients with Intracerebral Hemorrhage under Oral Anticoagulation

**DOI:** 10.3390/jcm9041077

**Published:** 2020-04-10

**Authors:** Sebastian Zimmer, Jörn Meier, Jens Minnerup, Moritz Wildgruber, Gabriel Broocks, Jawed Nawabi, Andrea Morotti, Andre Kemmling, Marios Psychogios, Uta Hanning, Peter B. Sporns

**Affiliations:** 1 Institute of Clinical Radiology, Westfaelische Wilhelms-University of Münster and University Hospital of Muenster, Albert-Schweitzer-Campus 1, Gebäude A1, 48149 Muenster, Germany; bam.zimmer@web.de (S.Z.); joernarnemeier@ukmuenster.de (J.M.); moritz.wildgruber@ukmuenster.de (M.W.); 2 Department of Medicine B, University Hospital Münster, 48149 Münster, Germany; 3 Department of Neurology, University Hospital of Muenster, Albert-Schweitzer-Campus 1, Gebäude A1, 48149 Muenster, Germany; minnerup@uni-muenster.de; 4 Department of Diagnostic and Interventional Neuroradiology, University Medical Center Hamburg-Eppendorf, Martinistr. 52, 20246 Hamburg, Germany; g.broocks@uke.de (G.B.); u.hanning@uke.de (U.H.); 5 Department of Radiology, Charité University Hospital, Charitéplatz 1, 10117 Berlin, Germany; jawed.nawabi@charite.de; 6 Neurology Unit, ASST Valcamonica, 25040 Esine (BS), Italy; andrea.morotti85@gmail.com; 7 Department of Neuroradiology, Westpfalz-Klinikum, 67655 Kaiserslautern, Germany; akemmling@gmail.com; 8 Department of Neuroradiology, Clinic of Radiology & Nuclear Medicine, University Hospital Basel, 4031 Basel, Switzerland; marios.psychogios@usb.ch

**Keywords:** intracerebral hemorrhage, outcome prediction, computed tomography

## Abstract

Introduction: In patients with spontaneous intracerebral hemorrhage (ICH), several non-contrast computed tomography (NCCT) markers and the spot sign (SS) in computed tomography (CT) angiography (CTA) have been established for the prediction of hematoma growth and neurological outcome. However, the prognostic value of these markers in patients under oral anticoagulation (ORAC) is unclear. We hypothesized that outcome prediction by these imaging markers may be significantly different between patients with and without ORAC. Therefore, we aimed to investigate the predictive value of NCCT markers and SS in patients with ICH under ORAC. Methods: This is a retrospective study of the database for patients with ICH at a German tertiary stroke center. Inclusion criteria were (1) patients with ICH, (2) oral anticoagulation within the therapeutic range, and (3) NCCT and CTA performed on admission within 6 h after onset of symptoms. We defined a binary outcome: modified Rankin Scale (mRS) ≤ 3 = good outcome versus mRS > 3 = poor outcome at discharge. The predictive value of each sign was assessed in uni- and multivariable logistic regression models. Results: Of 129 patients with ICH under ORAC, 76 (58.9%) presented with hypodensities within the hematoma in admission NCCT, 64 (52.7%) presented with an irregular shape of the hematoma, 60 (46.5%) presented with a swirl sign, 49 (38.0%) presented with a black hole sign, and 46 (35.7%) presented with a heterogeneous density of the hematoma. Moreover, 44 (34.1%) patients had a satellite sign, in 20 (15.5%) patients, an island sign was detected, 18 (14.0%) patients were blend-sign positive, and 14 (10.9%) patients presented with a CTA spot sign. Inter-rater agreement was very high for all included characteristics between the two readers. Multivariable logistic regression analysis identified the presence of black hole sign (odds ratio 10.59; *p* < 0.001), swirl sign (odds ratio 14.06; *p* < 0.001), and satellite sign (odds ratio 6.38; *p* = 0.011) as independent predictors of poor outcome. Conclusions: The distribution and prognostic value of several NCCT markers and CTA spot sign in ICH patients under ORAC is comparable to those with spontaneous ICH, even though these parameters are partly based on coagulant status. These findings suggest that a similar approach can be used for further research regarding outcome prediction in ICH patients under ORAC and those with spontaneous ICH.

## 1. Background and Purpose

Intracerebral hemorrhage (ICH) is a major cause of morbidity and mortality worldwide [1,2]. There are several factors directly associated with functional outcome such as admission hematoma location, hematoma volume, and admission blood pressure [1]. Together with these factors, hematoma growth presents an independent predictor of poor outcome [3,4,5,6], but in contrast to them, it is potentially modifiable if detected early enough and hence is an appealing therapeutic target [7,8]. 

The spot sign in computed tomographic angiography (CTA) is an established parameter for predicting hematoma growth and neurological outcome. However, only 11–25% of ICH patients undergo CTA during the acute phase in Western countries [9,10], and very few patients will undergo this test in low or middle-income countries. Moreover, contraindications such as allergic reactions to contrast medium as well as renal dysfunction and hyperthyroidism can pose a barrier to contrast administration. In this context, several non-contrast CT (NCCT) markers have been described as alternatives to the CTA spot sign to add discrimination to simple models for HE and outcome prediction [11,12]. For example, recent studies found an association of the appearance of the blend sign and a secondary neurological deterioration and showed that the blend sign and black hole sign predict poor neurological outcome [13,14]. In another multicenter series, the interaction of blend sign, hypodensities, black hole sign, and island sign in NCCT and the established spot sign in CTA and their individual contribution for prediction of neurological outcome were described [15]. The available evidence resulted in “International Standards for Detecting, Reporting and Interpreting Non-contrast computed tomography (CT) Markers of Intracerebral Hemorrhage Expansion” [16].

However, even though those studies prove that NCCT imaging markers and CTA spot sign can reliably predict a poor neurological outcome in patients with spontaneous ICH, patients with oral anticoagulant (ORAC)-related ICH were excluded or underrepresented in these studies. This means that there is no reliable evidence to date about how many patients with ORAC present with any of the reported NCCT signs. Compared to one-third of patients without anticoagulant treatment, the frequency of hematoma expansion is even higher in patients under oral anticoagulant therapy [4]. Most importantly, rates of reported NCCT signs may vary significantly between patients under ORAC therapy and those who are not, because these signs are to a great extent based on the hematoma heterogeneity and thereby the coagulant status of the ICH patients.

Therefore, we sought to evaluate the frequency of NCCT markers in ICH patients under ORAC and compare the rate of the most frequent markers to the results of previous studies including patients without anticoagulant therapy. We hypothesized that the frequency of the reported signs may vary significantly between the two patient groups.

## 2. Methods

### 2.1. Study Design

We retrospectively studied the radiological database of a German tertiary care center (University Hospital of Muenster, Münster, Germany) for patients aged >18 years with ICH confirmed on admission imaging admitted between January 2013 and December 2018.

Inclusion criteria were (1) ICH confirmed on NCCT, (2) anticoagulant treatment within therapeutic range (defined as INR ≥ 2 for patients with Marcumar) at time of ICH, and (3) NCCT and CTA performed on admission within 6 h after onset of symptoms. Patients were excluded if they had brain tumor, head trauma, vascular malformation, primary intraventricular hemorrhage, or ICH from hemorrhagic transformation of ischemic infarction.

We defined a binary outcome (good versus poor): Poor outcome was defined as modified Rankin Scale (mRS) >3 on discharge, good outcome was defined as mRS ≤ 3 [17]. Additionally, we obtained vascular risk factors (hypertension, diabetes mellitus) from patients’ medical charts. Conservative blood pressure management included the lowering of target systolic blood pressure below 140 mmHg. Patients with large hematomas underwent surgical evacuation according to local standards and at the discretion of the responsible neurosurgeon.

The study was approved by the local ethics committee. All study protocols and procedures were conducted in accordance with the Declaration of Helsinki.

### 2.2. Imaging Analysis

The CT scans were performed using standard clinical parameters with axial 5-mm section reconstruction thickness. The images were obtained and stored for further evaluation. Two experienced readers (both radiologists with 9 and 7 years of neuroradiological experience) independently evaluated the presence of NCCT imaging markers in all patients’ non-contrast CTs and then independently evaluated the presence of SS in the corresponding CTAs. Both readers were blinded to all clinical information. Discrepancies were settled by joint discussion. All signs were defined as recently published [16,18]; spot sign was defined according to the definition used in the PREDICT study [19].

### 2.3. Statistical Analysis

A univariable distribution of metric variables is described by median and interquartile range (IQR). Absolute and relative frequencies are given for categorical data. The Mann–Whitney *U* test or χ^2^ test was used to compare two independent samples on a metric or categorical outcome. In order to measure the inter-rater agreement, we used Cohen’s kappa.

Association between radiological and clinical parameters and outcome was assessed by logistic regression analysis. For multivariable model building, stepwise forward selection was used (inclusion criterion: *p*-value of the score test ≤0.05, exclusion criterion: *p*-value of the likelihood ratio test >0.1). Then, the factors of the model from step 1 were fitted together with all pairwise interactions in a second block using stepwise forward selection (inclusion: *p*-value of the score test ≤0.05 and exclusion: *p* value of the likelihood ratio test >0.1).

The variables considered for multivariable model building are given in Table 1. For selected variables, odds ratios with 95% confidence interval and *p* value of likelihood ratio tests are presented. Odds were calculated as the ratio of the probability for poor outcome to the probability for good outcome.

Analyses are regarded as explorative without adjustment for multiple testing. Local, unadjusted *p*-values ≤ 0.05 were considered as statistically noticeable.

Statistical analyses were performed in SPSS version 24 (IBM Corporation, Armonk, NY, USA).

## 3. Results

### 3.1. Baseline Characteristics

A total of 129 patients with a median age of 75 years (IQR: 65; 81) were included. Of these, 72 (55.8%) were female. Poor outcome (defined as mRS at discharge ≥ 3) was observed in 64 (49.6%) patients. Of these patients, 52 had intraventricular hemorrhage; however, there was no significant difference regarding outcomes between patients with and without secondary intraventricular hemorrhage (*p* = 0.095). Nineteen of the 129 patients (14.7%) were on novel oral anticoagulants (NOAC), and 110 (85.3%) received Marcumar. Moreover, we obtained vascular risk factors such as hypertension and diabetes mellitus as well as the frequency of surgical hematoma evacuation and conservative treatment (Table 1).

### 3.2. Poor Outcome and Interobserver Agreement

Of the 129 patients, 76 (58.9%) presented with hypodensities on admission NCCT, 64 (52.7%) presented with an irregular shape of the hematoma, 60 (46.5%) presented with a swirl sign, 49 (38.0%) presented with a black hole sign within the hematoma, and 46 (35.7%) presented with a heterogeneous density of the hematoma. Moreover, 44 (34.1%) patients had a satellite sign, in 20 (15.5%) patients, an island sign was detected, 18 (14.0%) patients were blend-sign positive, and 14 (10.9%) patients presented with a CTA spot sign. Inter-rater agreement was very high for all included characteristics between the two readers (Table 2).

The presence of hypodensities, a swirl sign, irregular shape, a heterogeneous density, black hole sign, blend sign, satellite sign, and island sign on NCCT (all < 0.001) as well as spot sign in the corresponding CTA (*p* = 0.020) were significantly associated with poor outcome. Notably, there was no significant association of the presence of fluid levels within the hematoma (*p* = 0.109) and additional intraventricular hemorrhage (*p* = 0.095) (Table 1).

Logistic regression analysis was performed to assess the association between the included parameters and poor outcome. In univariable logistic regression, the presence of hypodensities, a swirl sign, black hole sign, satellite sign, irregular shape, and heterogeneous density (all *p* < 0.001) as well as the presence of an island sign (*p* = 0.001), blend sign (*p* = 0.002), or spot sign (*p* = 0.032) on admission CT scan were associated with poor outcome (Table 3).

Multivariable logistic regression analysis confirmed the presence of a black hole sign (odds ratio 10.59; *p* < 0.001), swirl sign (odds ratio 14.06; *p* < 0.001), and satellite sign (odds ratio 6.38; *p* = 0.011) as independent predictors of poor outcome (Table 4).

## 4. Discussion

Our results show that several imaging signs that have been reported in patients with spontaneous ICH can similarly be observed in ICH patients under oral anticoagulation. In fact, in our study, any hypodensity within the hematoma—a swirl sign, an irregular shape, a heterogeneous density, black hole sign, blend sign, satellite sign and island sign on NCCT as well as spot sign in the corresponding CTA in univariable analysis—were all significantly associated with a poor outcome in ICH patients under ORAC.

As described by a recent expert consensus paper, NCCT imaging signs can be categorized into either ICH shape features (such as an island sign and satellite sign) or ICH density features (i.e., blend sign, black hole sign, hypodensities) [16,18]. We hypothesized that especially the distribution of ICH density features, which is based on coagulant status, might be influenced by a therapy with oral anticoagulants. However, the distribution of all signs in our study was comparable to previous studies including patients with spontaneous ICH [13,14,15,16,18,20]. Therefore, our study suggests that outcome prediction in ICH patients under oral anticoagulation can be made using the same markers that have been proven to be useful in patients with spontaneous ICH.

In our cohort, the strongest independent predictors of poor outcome were the presence of a black hole sign, swirl sign, or satellite sign on admission NCCT. However, as previously described, there is an important degree of overlap between several imaging characteristics [15,16], so future efforts should be directed toward creating easily applicable scoring systems to identify those patients at high risk for hematoma expansion and poor outcome. This effort may be supported by artificial intelligence and machine learning tools in the future. Our current study suggests that not only patients with spontaneous ICH but also patients under ORAC should be included in these efforts.

The limitations of our study can partly be attributed to its retrospective nature and relatively small sample size. Another limitation is the missing long-term follow up that might offer additional information but was not available for this study [5,6]. Moreover, in our study cohort, follow-up scans were not available in some patients before undergoing surgical hematoma evacuation. Therefore, we cannot draw conclusions on the frequency of hematoma expansion even though it is known to be directly associated with neurological outcome in patients with spontaneous ICH [7]. An advantage of our study is the availability of a CTA in all patients, which makes it possible to compare the presence of NCCT markers with the CTA spot sign.

## 5. Conclusions

The distribution and prognostic value of several NCCT markers and CTA spot sign in ICH patients under ORAC is similar to those with spontaneous ICH, even though these parameters are partly based on coagulant status. These findings suggest that a similar approach can be used for diagnostic workup and further research regarding outcome prediction in ICH patients under ORAC and those with spontaneous ICH.

## Figures and Tables

**Table 1:** Comparison of baseline demographic, clinical, and radiological characteristics between patients with and without poor outcome. IQR: interquartile range, mRS: modified Rankin Scale.

Baseline Clinical and Imaging Characteristics	All (*n* = 129)	Good Outcome (mRS ≤ 3) (*n* = 65)	Poor Outcome (mRS > 3) (*n* = 64)	*p*-Value
Age at admission (y), median (IQR)	75 (65;81)	71 (63; 79)	78 (69; 80)	0.141
Gender female, *n* (%)	72 (55.8%)	27 (47.4%)	24 (37.5%)	0.129
Hypertension, *n* (%)	69 (53.4%)	34 (52.3%)	35 (54.7%)	0.618
Diabetes mellitus, *n* (%)	16 (12.4%)	9 (13.8%)	7 (10.9%)	0.479
Intraventricular hemorrhage, *n* (%)	52 (40.3%)	21 (34.4%)	31 (49.2%)	0.095
Hypodensities positive, *n* (%)	76 (58.9%)	22 (33.8%)	54 (84.4%)	<0.001
Irregular shape positive, *n* (%)	64 (52.7%)	15 (23.1%)	53 (82.8%)	<0.001
Swirl sign positive, *n* (%)	60 (46.5%)	8 (12.3%)	52 (81.3%)	<0.001
Black hole sign positive, *n* (%)	49 (38.0%)	7 (10.8%)	42 (65.6%)	<0.001
Heterogeneous density, *n* (%)	46 (35.7%)	9 (13.8%)	37 (57.8%)	<0.001
Satellite sign positive, *n* (%)	44 (34.1%)	4 (6.2%)	40 (62.5%)	<0.001
Island sign positive, *n* (%)	20 (15.5%)	0 (0%)	43 (37.7%)	<0.001
Blend sign positive *n* (%)	18 (14.0%)	2 (3.1%)	16 (25.0%)	<0.001
Spot sign positive, *n* (%)	14 (10.9%)	3 (4.6%)	11 (17.2%)	0.020
Fluid level positive, *n* (%)	11 (8.5%)	3 (4.6%)	8 (12.5%)	0.109
Novel oral anticoagulants (NOAC)	19 (14.7%)	7 (10.8%)	12 (18.8%)	0.503
Apixaban, *n* (%)	8 (6.2%)	3 (37.5%)	5 (62.5%)	0.452
Dabigatran, *n* (%)	2 (1.5%)	0 (0%)	2 (100%)	0.151
Rivaroxaban, *n* (%)	9 (6.9%)	4 (44.4%)	5 (55.6%)	0.712
Marcumar, *n* (%)	110 (85.3%)	58 (52.7%)	52 (47.3%)	0.937
Anticoagulant reversal, *n* (%)	41 (37.3%)	18 (43.9%)	23 (56.1%)	0.769
Surgical hematoma evacuation, *n* (%)	27 (20.9%)	12 (44.4%)	15 (55.6%)	0.854
Blood pressure management, *n* (%)	102 (79.1%)	50 (49.0%)	52 (51.0%)	0.978

**Table 2:** Interrater Agreement for non-contrast computed tomography (NCCT) markers and computed tomographic angiography (CTA) spot sign.

Characteristics	kappa
Black hole sign	0.950
Blend Sign	0.900
Hypodensities	0.887
Island Sign	0.943
Spot sign	0.839
Swirl sign	0.968
Satellite sign	0.982
Fluid level	1.000
Irregular shape	0.905

**Table 3:** Univariable analysis of predictors of poor outcome.

Baseline Characteristics	OR	95% CI	*p*-Value
Age at admission (years)	1.02	0.99–1.05	0.123
Gender (ref: female)	1.72	0.85–3.5	0.130
Intraventricular hemorrhage (ref: no)	1.85	0.90–3.80	0.097
Black hole sign (ref: no)	15.82	6.19–40.44	<0.001
Blend sign (ref: no)	10.50	2.30–47.87	0.002
Hypodensities (ref: no)	10.56	4.52–24.65	<0.001
Island sign (ref: no)	31.00	4.02–239.36	0.001
Spot sign (ref: no)	4.29	1.14–16.2	0.032
Swirl sign (ref: no)	30.88	11.7–81.48	<0.001
Satellite sign (ref: no)	25.42	8.20–78.77	<0.001
Fluid level positive (ref: no)	2.95	0.75–11.68	0.123
Irregular shape positive (ref: no)	16.06	6.74–38.29	<0.001
Heterogeneous density (ref: no)	8.53	3.60–20.17	<0.001

Legend: Univariable analysis of predictors of poor outcome using logistic regression. Given are Odds Ratios (OR) with 95% confidence interval (CI) and *p*-value of likelihood ratio test if not otherwise specified. ICH: intracerebral hemorrhage CT: computed tomography.

**Table 4:** Multivariable analysis of predictors for poor outcome: the final model.

Baseline Characteristics	OR	95% CI	*p*-Value
Age at admission (years)	-	-	NS
Gender (ref: female)	-	-	NS
Intraventricular hemorrhage (ref: no)	-	-	NS
Black hole sign (ref: no)	10.59	2.95–38.16	<0.001
Blend sign (ref: no)	-	-	NS
Hypodensities (ref: no)	-	-	NS
Island sign (ref: no)	-	-	NS
Spot sign (ref: no)	-	-	NS
Swirl sign (ref: no)	14.06	4.14–47.78	<0.001
Satellite sign (ref: no)	6.38	1.52–26.83	0.011
Fluid level positive (ref: no)	-	-	NS
Irregular shape positive (ref: no)	-	-	NS
Irregular shape positive (ref: no)	-	-	NS
Heterogeneous density (ref: no)	-	-	NS

Legend: Multivariable analysis of predictors of poor outcome using stepwise forward selection in logistic regression (for details, see Methods). Given are Odds Ratios (OR) with 95% confidence interval (CI) and *p*-value of likelihood ratio test for selected variables. For non-selected variables, a *p*-value of the score test is displayed. No interaction terms were selected. N/S: not selected; ICH: intracerebral hemorrhage; CT: computed tomography.
